# Regulatory non-coding somatic mutations as drivers of neuroblastoma

**DOI:** 10.1038/s41416-025-02939-0

**Published:** 2025-01-23

**Authors:** Annalaura Montella, Matilde Tirelli, Vito Alessandro Lasorsa, Vincenzo Aievola, Vincenza Cerbone, Rosa Manganiello, Achille Iolascon, Mario Capasso

**Affiliations:** 1 University of Naples Federico II, Department of Molecular Medicine and Medical Biotechnology, Naples, Italy; 2 CEINGE Biotecnologie Avanzate Franco Salvatore, Naples, Italy; 3 Present Address: University of Naples Federico II, Department of Molecular Medicine and Medical Biotechnology, Naples, Italy

**Keywords:** Cancer genetics, Functional genomics

## Abstract

**Background:**

Emerging evidence suggests that non-coding somatic single nucleotide variants (SNVs) in *cis*-regulatory elements (CREs) contribute to cancer by disrupting gene expression networks. However, the role of non-coding SNVs in cancer, particularly neuroblastoma, remains largely unclear.

**Methods:**

SNVs effect on CREs activity was evaluated by luciferase assays. Motif analysis and ChIP-qPCR experiments were employed to reveal the transcription factors (TFs) involved in these processes. We exploited CRISPR-Cas9 experiments to elucidate the role of these SNVs on the CREs target genes expression. Cell proliferation and invasion assays were performed to assess their role in neuroblastoma tumorigenesis.

**Results:**

Our findings demonstrate that non-coding SNVs modify the transcriptional activity of two CREs altering the binding of STAT3 and SIN3A. Therefore, these SNVs reduce the expression of *CTTNBP2* and *MCF2L*. We demonstrate that these two genes act as tumor suppressor in neuroblastoma. These pathogenetic SNVs may serve as oncogenic drivers by impairing the transcriptional programs essential for neuronal development and differentiation in which both the investigated TFs and target genes are involved.

**Conclusion:**

Overall, the understanding of the functional role of non-coding variants elucidates their impact on tumorigenesis and can uncover new potential targets of cancer therapeutic strategies.

## Introduction

Despite advancements in understanding the molecular mechanisms underlying cancer development, the role of genetic variations, especially those within the non-coding regions of the genome, remains an area of intense research and interest. Single nucleotide variants (SNVs) in non-coding regions have emerged as critical elements that can influence gene expression and, consequently, cellular function and behavior [1, 2]. Unlike mutations in coding regions that directly alter protein function, non-coding SNVs can influence gene expression by affecting cis-regulatory elements (CREs) such as promoters and enhancers thus contributing indirectly to cancer development [3]. For instance, a landmark study identified a non-coding SNV in the *TERT* promoter region, significantly increasing telomerase activity in melanoma, underscoring the potential of non-coding SNVs to drive cancer progression [4]. Similarly, another study highlighted non-coding SNVs within enhancer elements of the *KRAS* and *PER2* oncogenes which resided in proximity to nuclear receptor binding regions and modulated transcriptional activities in response to nuclear receptor signaling in leukemia cells [5].

Our recent investigations have further highlighted the significance of non-coding somatic mutations in neuroblastoma (NB), revealing a substantial enrichment of SNVs in transcription factor binding sites of regulatory elements specifically active in NB. These variants are postulated to influence disease initiation by altering gene expression and regulatory networks [6]. In particular, we identified three clusters of SNVs which were predicted to impact the expression of genes, already known to be correlated with prognostic markers in other cancer types [6]. Additionally, we have identified somatic SNVs in active CREs that impact crucial developmental and immune response genes, which are associated with poor survival in NB [7]. For instance, an excess of somatic SNVs in a CRE predicted to interact with *IPO7* were identified and functionally investigated, confirming that these variants actually alter the expression of this gene [7]. Altogether, these studies highlight the critical impact of non-coding genetic variations on NB pathogenesis and prognosis.

Despite these insights, the precise mechanisms through which non-coding SNVs contribute to cancer are not yet fully understood, highlighting the need for focused research to decipher the intricate relationship between non-coding genetic variants and cancer pathogenesis. The current gap in our comprehension is partially due to the challenges associated with conducting in vitro functional analyzes of SNVs located within intricate genomic landscapes [8].

NB, a common pediatric cancer originating from the sympathetic nervous system, presents an ideal model for investigating the impact of non-coding genetic variations due to its diverse genetic background and the significant role that gene regulation plays in its development and progression [9]. Currently, NB remains a lethal disease in approximately half of high-risk patients, even with aggressive treatment modalities [10]. Based on clinically different pretreatment groups, NB patients are classified in very low, low, intermediate, and high-risk groups in terms of 5-year event-free survivals. This classification is based on risk factors included into the International Neuroblastoma Risk Group Staging System (INRG). The INRG classification system includes stage, age, histology, tumor grade, *MYCN* status, 11q alteration status, and DNA ploidy [11]. Amplification of the *MYCN* oncogene is one of the most critical genomic markers used to categorize NB risk, often associated with a poor prognosis [12]. Moreover, other chromosomal aberrations, including deletions of 1q and 11q, gains of 17q, rearrangements at the *TERT* locus, and the combination of 19p loss with 1q gain in older children, have been recognized as indicators of poor outcomes [12–14]. Extensive genetic investigations have identified numerous germline variants, both common and rare, that are implicated in NB susceptibility [15–17]. Functional studies of some of the common variants in non-coding regions have demonstrated their role in regulating gene expression involved in NB tumorigenesis [18–21]. Of note, rs17489363 at the *BARD1* locus was found to be associated with predisposition to high-risk NB and correlated with low expression of full-length *BARD1* which may act as tumor suppressor [18].

In contrast, somatic sequencing efforts have typically revealed a paucity of mutations that drive cancer, with notable exceptions being the activating mutations in the *ALK* gene and inactivating mutations in the *ATRX* gene [22]. Our recent research has brought to light the relatively rare N546K mutation in the *FGFR1* gene, which activates downstream signaling pathways, culminating in an aggressive tumor phenotype and resistance to FGFR1 inhibitors [23]. However, much of this research has focused on the coding genome, despite whole-genome sequencing studies suggesting that numerous genetic alterations within non-coding regulatory regions are equally capable of contributing to cancer development [1, 3].

In this study, we investigate and clarify the mechanisms by which non-coding somatic SNVs cause deregulation of CREs activity and consequently the expression of target genes, thus contributing to NB initiation and progression. Specifically, we have carried out an in-depth functional analysis of non-coding somatic SNVs in NB, previously predicted to regulate Cortactin-Binding Protein 2 (CTTNBP2) and MCF.2 Cell Line Derived Transforming Sequence Like (MCF2L*)* genes, as reported in our prior study [6].

## Material and Methods

### In silico gene expression analysis

From the Cancer Dependency Map (DepMap) portal, the expression levels of *CTTNBP2* and *MCF2L* were assessed in NB cell lines per data available in the Expression public 22Q4 release. The R2: Genomics Analysis and Visualization platform (available at http://r2.amc.nl) provided analytical tools to investigate the association between the expression of *CTTNBP2* and *MCF2L* and NB prognostic markers, including *MYCN* amplification, disease stage, and age at diagnosis. This analysis was performed using RNA expression data from a cohort of 105 primary NB tumors, as cataloged in the NCBI GEO database (accession: GSE73517) [24]. In particular, after selecting the dataset of interest, we used the “datagrabber” function of the R2 platform which allows users to download both gene expression data and clinical characteristics of selected genes. Subsequently, we used the R software for statistical computing to perform association analyzes, obtain plots and calculate statistical significance by Wilcoxon-Mann-Whitney test.

### In vitro evaluation of genes protein and mRNA levels

Cell lines used for in vitro studies were cultured as detailed in Supplementary Materials. The expression levels of *CTTNBP2, MCF2L, STAT3, SIN3A* and *β-actin* mRNA and protein levels were evaluated using quantitative PCR (qPCR) and western blotting, respectively, as previously published [25]. Densitometry analysis performed by ImageJ software [26], primary antibodies and specific primers were detailed in Supplementary Materials.

### Luciferase reporter gene assay

Luciferase assays to evaluate the role of somatic non-coding variants on CREs activity were carried out as previously reported [7]. See Supplementary Materials for additional information.

### Gene silencing

STAT3, SIN3A, CTTNBP2 and MCF2L-specific siRNA-27 duplexes (pooled siRNA A-B-C) and SR30004-Universal Scrambled Negative Control siRNA (DS Scrambled Neg) duplex (Origene) were used at a concentration of 10 nM to transfect LAN-2, SH-SY5Y, CHP-212 and SK-N-BE(2) cells with X-tremeGENE siRNA Transfection Reagent (Roche). After 24 h, 48 h and 72 h of transfection, cells were harvested to assess the silencing of protein and mRNA of above reported genes.

Gene silencing in combination with luciferase assays was evaluated after 72 h from plasmid siRNA transfection.

### Prediction of altered transcription factor binding motifs by motifbreakeR

Motif analysis with the R-Bioconductor package “motifbreakeR” [27] was performed to assess whether the somatic variants within the selected CREs altered TF binding motifs. The ENCODE database of PWMs (Positional Weighted Matrices) was used as a reference. Motif breaks or gains were considered neutral if the difference of binding efficiency was within ± 0.2.

### ChIP-qPCR experiments

Chip-qPCR experiments on CTTNBP2 CRE ChIP (SNV1), CTTNBP2 CRE ChIP (SNV2) and MCF2L CRE ChIP plasmids containing the wild-type and the mutant sequence used in the luciferase reporter assays were performed according to Zhou S, et al. [28]. The procedure is detailed in Supplementary Materials.

### CRISPR-Cas9 experiments

To mimic SNVs effects on expression of their predicted target genes, two different strategies of CRISPR-Cas9 genome editing were performed. For chr7:117513318:G > C or CTTNBP2 CRE SNV1, insertions and deletions (INDELs) mimicking the SNV were introduced into the HEK-293 cell line as previously described [7], while for MCF2L CRE, deletion of a region which contains both chr13:114058179:T > C or MCF2L CRE SNV1 and chr13:114058184:G > A or MCF2L CRE SNV2 was performed in SK-N-BE(2) cells. Genomic coordinates are based on the GRCh37/hg19 assembly of the human genome. Details are reported in Supplementary Materials.

### In-house HiC analysis

In-house HiC data of the SK-N-BE(2) cell line were processed as reported previously [29]. After sequencing using an Illumina HiSeq platform, paired-end reads of 150 base pairs (bp) were aligned to the reference genome (build hg19/GRCH37) using Bowtie2. HiCExplorer (v3.7) was used to (1) build the interaction matrix at a resolution of 10 Kb (bin size = 10 Kb), (2) normalize the observed read counts, (3) determine topologically associating domains (TADs, self-interacting genome regions) and their boundaries, and (4) plot the results. Subsequently, starting from MCF2L CREs cluster of variants coordinates (chr13:114058178-114058287), we extended our region of interest of 0.5 Mb up- and downstream and calculated the statistical significance of the interactions between bins with FitHiC (v2.0.8). p-values were corrected for multiple tests using the Benjamini-Hochberg method (false discovery rate, FDR), and the cutoff was set at 5%. Finally, we annotated these bins using the R-Bioconductor package ChIPseeker (v 1.32.0) to map genomic bins to gene coordinates.

### Cell viability and invasion assays

In NB cells silenced for *CTTNBP2* and *MCF2L*, cell viability (0 h - 24 h - 48 h - 72 h) and invasion capability (48 h) were assessed as previously described [23]. For edited clones the same procedure was used after 24 h from seeding.

## Results

### SNVs in cis-regulatory elements of CTTNBP2 and MCF2L alter the transcriptional regulatory activity

To investigate the functional significance of somatic non-coding variants in NB, we assessed the role of putative pathogenetic variants identified through a computational analysis in our previous study [6], which have not been functionally explored before.

In particular, we focused on the effects of two variants located in the first intron and nearby the transcription starting site of the *CTTNBP2* gene, respectively: chr7:117513318:G > C (CTTNBP2 CRE SNV1) and chr7:117513582:G > T (CTTNBP2 CRE SNV2). Additionally, we examined two variants, chr13:114058179:T > C (MCF2L CRE SNV1) and chr13:114058184:G > A (MCF2L CRE SNV2), located within a CRE predicted to interact with *MCF2L* [6].

First, we selected NB cell lines characterized by high expression levels of *CTTNBP2* and *MCF2L*. Using the public DepMap database (https://depmap.org/portal/), our analysis revealed that most NB cell lines exhibit low expression of *CTTNBP2*, with the LAN-2 cell line showing the highest expression levels (Supplementary Fig. 1A). In contrast, the expression of *MCF2L* differed across NB cell lines, with SK-N-BE(2) and CHP-212 displaying among the highest levels (Supplementary Fig. 1B). Subsequently, we validated these observations by measuring the protein and mRNA levels of *CTTNBP2* and *MCF2L* in different NB cell lines (Supplementary Fig. 1C). Therefore, we chose NB cell lines that exhibited the highest levels of CREs target genes for our experimental investigations. Specifically, given that SH-SY5Y cells displayed at least detectable levels of CTTNBP2 protein and mRNA (Supplementary Fig. 1C), we selected this cell line together with LAN-2 for the evaluation of CTTNBP2 CRE SNV1 and CTTNBP2 CRE SNV2. Instead, for the analysis of MCF2L CRE SNV1 and MCF2L CRE SNV2, we used the SK-N-BE(2) and CHP-212 cell lines. In the selected NB cell lines, we performed luciferase assays on each single SNV to evaluate their impact on the regulatory activity of their corresponding CRE. Our results revealed that CTTNBP2 CRE SNV1 (in red in Supplementary Fig. 2A), MCF2L CRE SNV1 and MCF2L CRE SNV2 (in light blue and green in Supplementary Fig. 2B, respectively) significantly reduced the activity of their respective regulatory elements. These findings agree with the predictions from our previously published *in-silico* analysis [6].

In contrast, CTTNBP2 CRE SNV2 (in purple in Supplementary Fig. 2A) exhibited an increased regulatory activity of CRE in SH-SY5Y cells, showing an opposing effect compared to the one observed in LAN-2 cells (Supplementary Fig. 2A).

Since promoter and enhancer regions are typically short, ranging from 100 to 1000 base pairs in size [30], we performed luciferase assays on smaller constructs to provide additional evidence regarding the regulatory effects of the following variants: CTTNBP2 CRE SNV1 (in red), CTTNBP2 CRE SNV2 (in purple), MCF2L CRE SNV1 (in light blue) and MCF2L CRE SNV2 (in green) (Supplementary Fig. 3A, B). All tested SNVs confirmed their regulatory function, except for MCF2L CRE SNV2, which did not significantly impact the transcriptional activity of MCF2L CRE (Supplementary Fig. 3A, B). Consequently, we have chosen to exclude it from further evaluation experiments.

Altogether, these results indicate that CTTNBP2 CRE SNV1 and MCF2L CRE SNV1 may potentially interfere with the binding of a transcriptional activator or enhance the binding with a repressor. On the other hand, CTTNBP2 CRE SNV2 exhibits a mechanism similar to the previous SNVs in LAN-2, while this variant shows an opposite effect in SH-SY5Y, possibly by decreasing the binding of a repressor or increasing the affinity for an activator.

### SNVs affect the binding to transcription factors involved in regulation of CTTNBP2 and MCF2L expression

We used motifbreakR tool [27] to investigate the potential effects of non-coding somatic SNVs on TF binding.

**Fig. 1:**
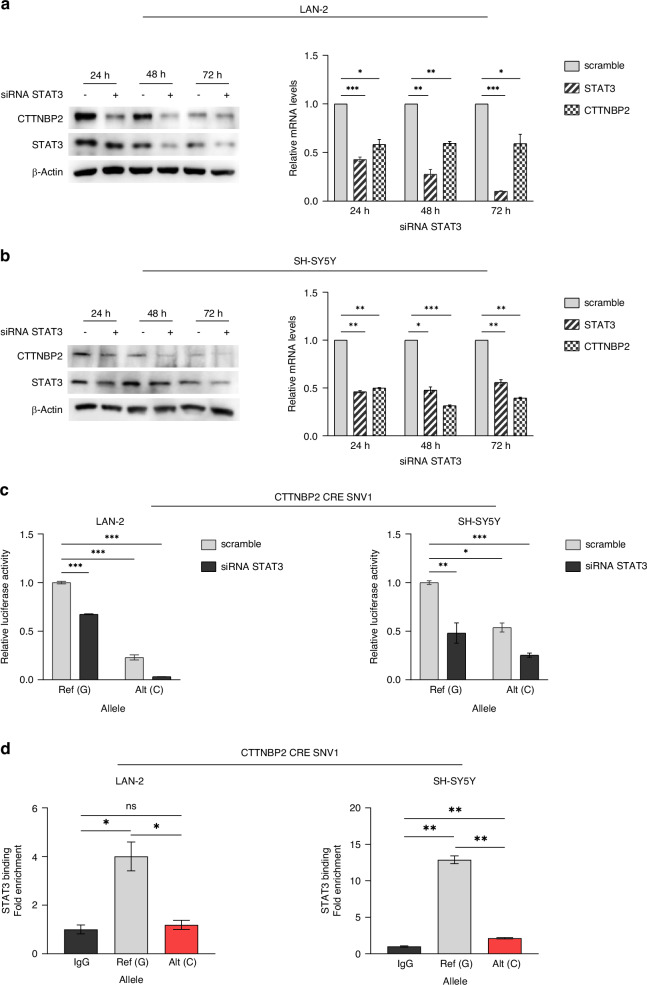
CTTNBP2 CRE SNV1 reduces the binding affinity between CTTNBP2 CRE and STAT3 which acts as transcriptional activator of CTTNBP2 gene. Correlation between STAT3 and CTTNBP2 expression in **a** LAN-2 and **b** SH-SY5Y cells transfected with three pooled STAT3 siRNA. CTTNBP2 and STAT3 expression relative to their respective siScrambles (represented as single scramble bar in histogram), as measured by western blot (left) and qPCR (right) 24 h 48 h and 72 h post STAT3 silencing. β-Actin protein levels are used as loading control. **c** Luciferase reporter gene assay for CTTNBP2 CRE SNV1 carried out in LAN-2 and SH-SY5Y 72 h post STAT3 silencing. Luciferase activity of CTTNBP2 CRE SNV1 is normalized to that from cells transfected with wild-type construct and siScramble (scramble). **d** Allele-specific ChIP-qPCR conducted on plasmids carrying the wild-type or CTTNBP2 CRE SNV1 sequence upon transient transfection in LAN-2 and SH-SY5Y cell lines. Data is presented as fold-change of variant sequence upon comparing to wild-type sequence. All data shown are the mean ± standard deviation from three independent experiments each done in triplicate. Significant *p*-values obtained by two-tailed T-test are reported by * (* < 0.05; ** <0. 01; *** <0.001). Ref Reference, Alt Altered.

Since the proposed mechanism of CTTNBP2 CRE SNV1 was to decrease a binding for a transcriptional activator, among the TFs reported by motifbreakR tool (Supplementary Table 1), we selected STAT3 because of its well-known role in several cancers [31]. To confirm the regulatory role of STAT3 as activator on *CTTNBP2* expression, we performed *STAT3* silencing in LAN-2 (Fig. 1a and Supplementary Fig. 4A) and SH-SY5Y (Fig. 1b and Supplementary Fig. 4A), which resulted in a decrease of CTTNBP2 protein and mRNA levels.

To assess the effective binding of STAT3 with CTTNBP2 CRE, we performed luciferase assays in NB cell lines in combination with *STAT3* silencing, using both long (Fig. 1c) and short constructs (Supplementary Fig. 5A). Our results indicated that *STAT3* downregulation reduced the transcriptional activity of the wild-type CTTNBP2 CRE, mimicking the effect of CTTNBP2 CRE SNV1 on the regulatory element. Furthermore, *STAT3* downregulation enhanced the transcriptional effect of the altered allele (C) on CTTNBP2 CRE activity (Fig. 1c and Supplementary Fig. 5A).

Then, we confirmed that CTTNBP2 CRE SNV1 decreased the binding affinity between STAT3 and CTTNBP2 CRE by performing ChIP-qPCR in both LAN-2 and SH-SY5Y cells (Fig. 1d).

Our findings suggest that CTTNBP2 CRE SNV1 decreased regulatory activity of CTTNBP2 CRE by reducing the affinity with STAT3, which acts as transcriptional activator of *CTTNBP2* in NB cell lines.

From motifbreakR tool, the second investigated somatic noncoding variant, CTTNBP2 aCRE SNV2, was predicted to decrease the binding for the repressor SIN3A (Supplementary Table 2). This finding was in line with the effect of this variant on regulatory element activity in SH-SY5Y (Supplementary Figs. 2A and 3A) and with our previous *in-silico* prediction [6].

**Fig. 2:**
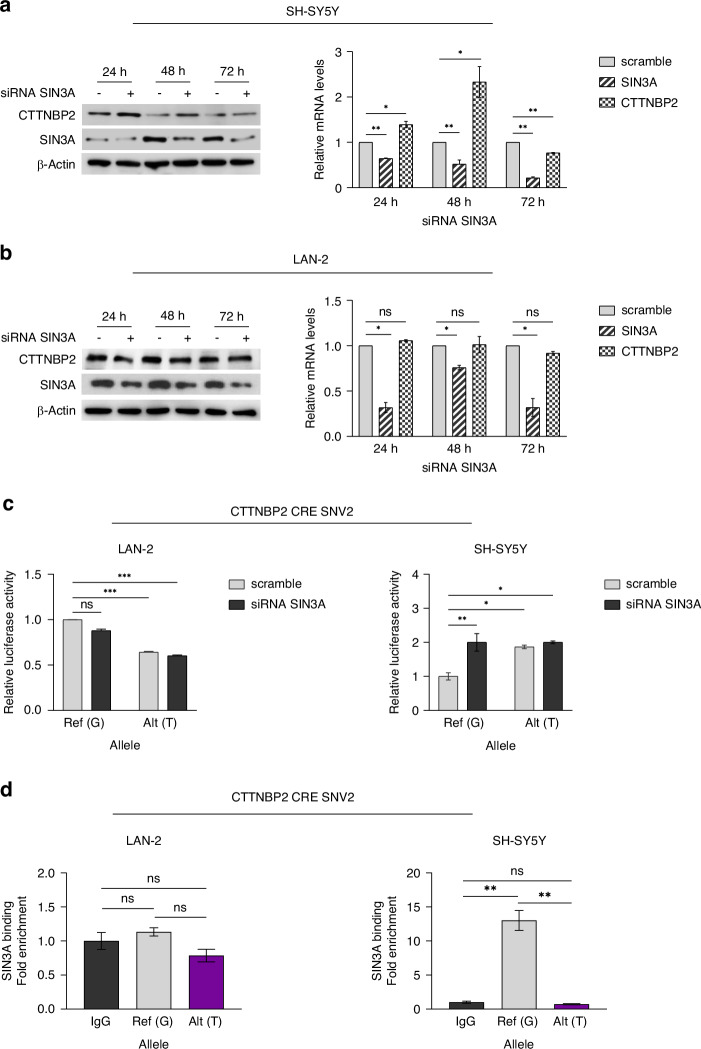
CTTNBP2 CRE SNV2 reduces the binding affinity between CTTNBP2 CRE and SIN3A which acts as transcriptional repressor of CTTNBP2 in SH-SY5Y cells. Correlation between SIN3A and CTTNBP2 expression in **a** LAN-2 and **b** SH-SY5Y transfected with three pooled SIN3A siRNA. CTTNBP2 and SIN3A expression relative to their respective siScrambles (reported as single scramble bar in histogram), as measured by western blot (left) and qPCR (right) 24 h, 48 h and 72 h post SIN3A silencing. β-Actin protein levels are used as loading control. **c** Luciferase reporter gene assay for CTTNBP2 CRE SNV2 carried out in LAN-2 and SH-SY5Y 72 h post SIN3A silencing. Luciferase activity of CTTNBP2 CRE SNV2 is normalized to that from cells transfected with wild-type construct and siScramble (scramble). **d** Allele-specific ChIP-qPCR conducted on plasmids carrying the wild-type or CTTNBP2 CRE SNV2 sequence upon transient transfection in LAN-2 and SH-SY5Y cell lines. Data is presented as fold-change of variant sequence upon comparing to wild-type sequence. All data shown are the mean ± standard deviation from three independent experiments, each done in triplicate. Significant *p*-values obtained by two-tailed T-test are reported by * (* < 0.05; ** <0.01; *** <0.001). Ns non-significant, *p*-value, Ref Reference, Alt Altered.

*SIN3A* silencing in SH-SY5Y cells led to an upregulation of CTTNBP2 protein and mRNA levels, suggesting that SIN3A likely functions as a transcriptional repressor of *CTTNBP2* in this cellular context (Fig. 2a and Supplementary Fig. 4B).

However, *SIN3A* downregulation in LAN-2 cells did not alter *CTTNBP2* expression (Fig. 2b and Supplementary Fig. 4B), indicating the involvement of a different TF in the impairment of CTTNBP2 CRE regulatory activity in presence of this variant (Supplementary Figs. 2A and 3A).

Luciferase assays in combination with *SIN3A* silencing in LAN-2 cell line confirmed that this TF did not bind CTTNBP2 CRE. In line with this, the silencing of *SIN3A* had no significant effect on the transcriptional activity of both the long and short wild-type CTTNBP2 CRE constructs in these cells (Fig. 2c and Supplementary Fig. 5B).

On the other hand, luciferase assays in SH-SY5Y cells silenced for *SIN3A* revealed an increase in the transcriptional activity of wild-type CTTNBP2 CRE, similarly to the effect of CTTNBP2 SNV2. Moreover, SIN3A silencing further enhanced the regulatory impact of this variant on CTTNBP2 CRE in SH-SY5Y (Fig. 2c and Supplementary Fig. 5B). Together these results imply that SIN3A interacts with CTTNBP2 CRE in SH-SY5Y cells, whereas such an interaction does not occur in LAN-2 cells. In this context, we suggest that the observed difference in SIN3A interaction with the CTTNBP2 CRE between SH-SY5Y and LAN-2 may be due to SIN3A’s role as a corepressor of REST in the repression of neuronal genes, potentially influencing *CTTNBP2* gene repression specifically in *MYCN* non-amplified NB cells, such as SH-SY5Y, where this transcription factor is overexpressed [32–34].

In addition, ChIP-qPCR experiments in both cell lines demonstrated that the CTTNBP2 CRE SNV2 reduced the binding of SIN3A in SH-SY5Y cells, while no alteration was detected in LAN-2 cells (Fig. 2d).

**Fig. 3:**
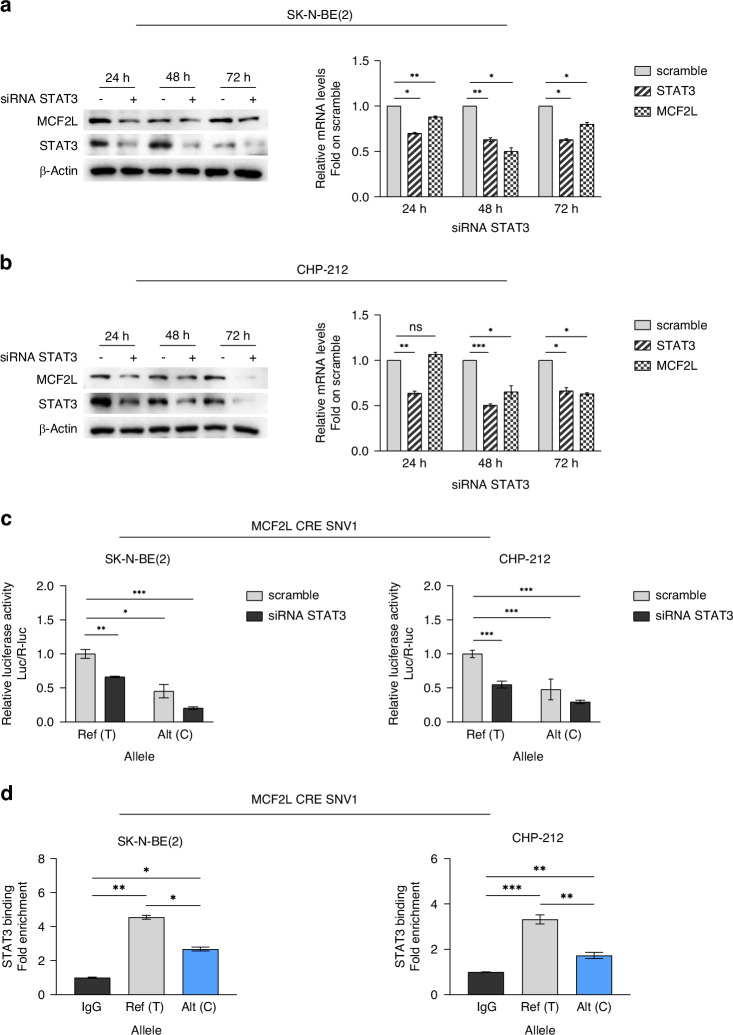
MCF2L CRE SNV1 decreases the binding affinity between MCF2L CRE and STAT3 which acts as transcriptional activator of MCF2L gene. Correlation between STAT3 and MCF2L expression in **a** SK-N-BE(2) and **b** CHP-212 cells transfected with three pooled STAT3 siRNA. MCF2L and STAT3 expression relative to their respective siScramble (reported as a single scramble bar in histogram), as measured by western blot (left) and qPCR (right) 24 h, 48 h and 72 h post STAT3 silencing. β-Actin protein levels are used as loading control. **c** Luciferase reporter gene assay for MCF2L CRE SNV1 carried out in SK-N-BE(2) and CHP-212 72 h post STAT3 silencing. Luciferase activity of MCF2L CRE SNV1 is normalized to that from cells transfected with wild-type construct and siScramble (scramble). **d** Allele-specific ChIP-qPCR conducted on plasmids carrying the wild-type or MCF2L CRE SNV1 sequence upon transient transfection in SK-N-BE(2) and CHP-212 cell lines. Data is presented as fold-change of variant sequence upon comparing to wild-type sequence. All data shown are the mean ± standard deviation from three independent experiments, each done in triplicate. Significant *p*-values obtained by two-tailed T-test are reported by * *(* < 0.05; ** < 0.01; *** < 0.001). Ns non-significant *p*-value, Ref Reference, Alt Altered.

The motifbreakR tool identified the third somatic non-coding variant studied, MCF2L CRE SNV1, as impacting the binding of different STAT family members (Supplementary Table 3). Accordingly, we focused on STAT3 as the TF implicated in the reduced activity of MCF2L CRE due to MCF2L SNV1. Silencing of *STAT3* in NB cell lines confirmed its role as an activator of *MCF2L* expression (Fig. 3a, b and Supplementary Fig. 4C). Then, we assessed the interaction between STAT3 and the MCF2L CRE by performing luciferase assays on both longer (Fig. 3c) and shorter (Supplementary Fig. 5C) constructs containing the regulatory element in NB cells silenced for *STAT3*.

These results showed that *STAT3* silencing diminished the normal transcriptional activity of MCF2L CRE and enhanced the impact of MCF2L CRE SNV1 (Fig. 3c and Supplementary Fig. 5C). Furthermore, ChIP-qPCR experiments confirmed the variant effect on STAT3 binding, showing a reduced interaction with MCF2L CRE (Fig. 3d). Similarly to CTTNBP2 CRE SNV1, MCF2L CRE SNV1 was found to decrease the regulatory activity of the MCF2L CRE by altering its interaction with STAT3, which functions as a transcriptional activator of *MCF2L* in NB cell lines.

### CTTNBP2 CRE SNV1 decreases the expression of *CTTNBP2*, which in turn leads to an increase in cell proliferation and invasion capability

To provide evidence that *CTTNBP2* constitutes a target gene of CTTNBP2 CRE and that CTTNBP2 CRE SNV1 downregulates its expression, we employed CRISPR-Cas9 genome editing in HEK-293 cells to introduce random INDELs near the SNV position.

We selected HEK-293 cells because they exhibit higher *CTTNBP2* levels (Supplementary Fig. 1C) compared to NB cells. In addition, HEK-293 cells are a commonly employed choice for genome editing experiments due to their high cell growth capability at single cell level. We did not perform CRISPR-Cas9 genome editing on LAN-2, which represented the only NB cell characterized by detectable *CTTNBP2* expression level because this cell line was not well-suited for single cell growth.

Through Sanger sequencing and the Inference of CRISPR Edits (ICE) analysis, we identified over 50% of clones as edited. We examined three clones to investigate the in vitro biological impact of CTTNBP2 CRE SNV1: clone gRNA1#1 (98% editing), clone gRNA1#7 (97% editing) and clone gRNA2#1 (97% editing) (Supplementary Fig. 6).

**Fig. 4:**
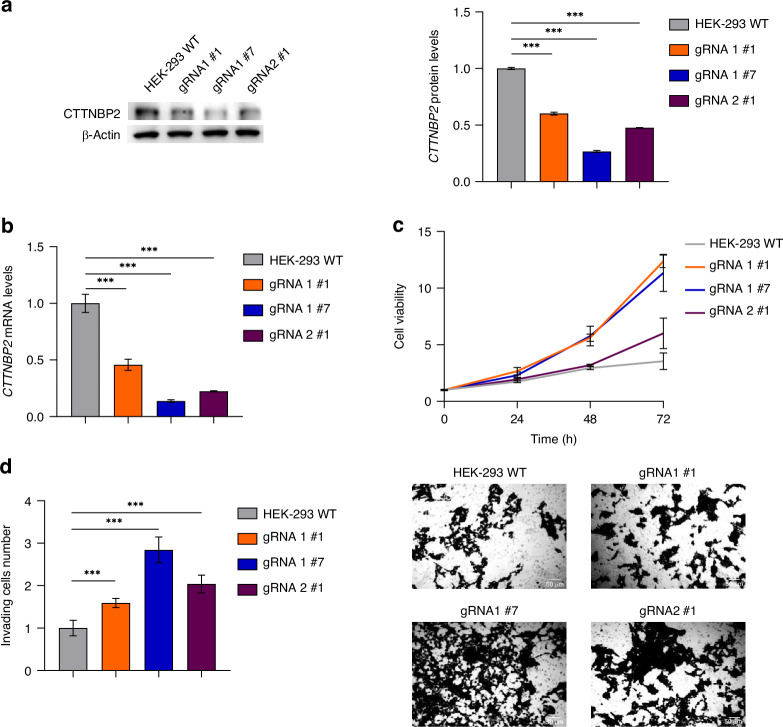
CTTNBP2 CRE SNV1 leads to decreased expression levels of CTTNBP2, which is associated with an enhancement of cell proliferation and the invasion capability. In edited clones, CTTNBP2 expression relative to HEK-293 wild-type cells, as measured by western blot **a** and qPCR **b**. β-Actin protein levels are used as loading control. **c** Cell viability and **d** invading cell number in edited clones is shown as fold change compared to wild-type cells. Representative images (10X) of invasion assay in edited clones are reported on the right. All data shown are the mean ± standard deviation from three independent qPCR experiments, each done in triplicate. Significant *p*-values obtained by two-tailed T-test are reported by * (* < 0.05; ** < 0.01; *** < 0.001).

Across all edited clones, a reduction in CTTNBP2 protein and mRNA levels was observed (Fig. 4a, b), alongside an increase in both cell proliferation (Fig. 4c) and invasion capability (Fig. 4d) relative to wild-type cells.

In summary, the CTTNBP2 CRE SNV1 variant leads to reduced expression of *CTTNBP2* which appears to enhance the tumorigenic phenotype in HEK-293 cells, indicating a potential role for *CTTNBP2* as a novel tumor suppressor.

For CTTNBP2 CRE SNV2, CRISPR-Cas9 genome editing was not pursued since the variant location was not suitable to gRNA design. Additionally, deletion of the region containing the variant was not feasible due to the proximity of an exonic region of the *CTTNBP2* gene.

### The deletion of MCF2L CRE decreases *MCF2L* expression and enhances cell proliferation and invasion capability

Before performing CRISPR-Cas9 genome editing in SK-N-BE(2) cells, we analyzed in-house HiC data obtained from this cell line to provide further evidence about the interaction between *MCF2L* and MCF2L CRE (Supplementary Fig. 7). Since MCF2L CRE SNV1 fell in a region unsuitable to introduce random INDELs around the variant position, we decided to delete the intergenic regulatory region containing this SNV to demonstrate the importance of the MCF2L CRE in modulating the expression of *MCF2L* gene.

In SK-N-BE(2), we targeted MCF2L CRE with two gRNA pairs that efficiently deleted the region overlapping the regulatory element (Supplementary Fig. 8A). PCR experiments, using primers flanking the targeted MCF2L CRE deletion, were performed to identify edited clones (Supplementary Fig. 8B).

**Fig. 5:**
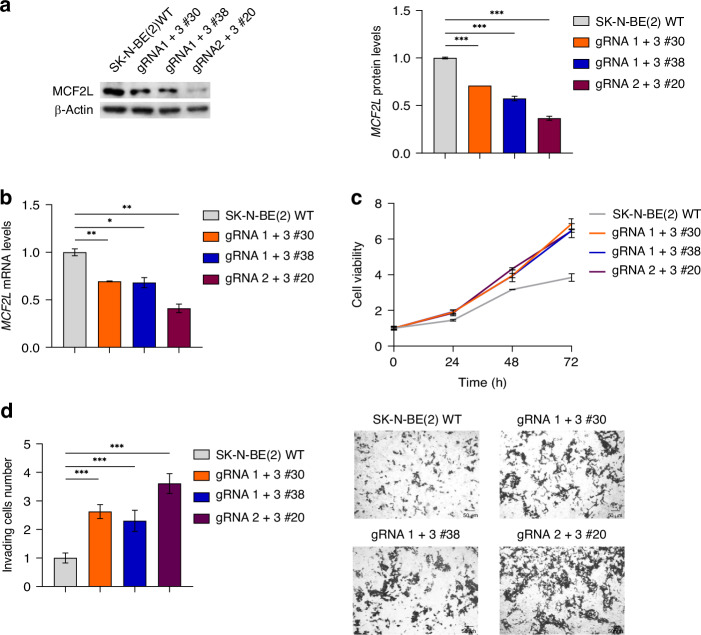
The deletion of the MCF2L CRE leads to decreased expression of *MCF2L* and increased cellular proliferation and invasion capacity. In edited clones, *MCF2L* expression relative to SK-N-BE(2) wild-type cells, as measured by western blot **a** and qPCR **b**. β-Actin protein levels are used as loading control. **c** Cell viability in edited clones is shown as fold change compared to SK-N-BE(2) wild-type cells. **d** Invading cells number in edited clones is shown as fold change compared to SK-N-BE(2) wild-type cells. Representative images (10×) of invasion assay in edited clones are reported on the right. All data shown are the mean ± standard deviation from three independent qPCR experiments, each done in triplicate. Significant *p*-values obtained by two-tailed T-test are reported by * (* < 0.05; ** < 0.01; *** < 0.001).

To assess if a deletion of MCF2L CRE modulated *MCF2L*, we evaluated its expression in edited clones. We observed that both heterozygotes (gRNA 1 + 3#30 and gRNA 1 + 3#38) and homozygote (gRNA 2 + 3#20) clones exhibited lower *MCF2L* expression levels compared to wild-type (Fig. 5a, b).

In addition, these clones showed an increase in cell proliferation (Fig. 5c) and cell invasion capability (Fig. 5d), suggesting a potential role of *MCF2L* in tumorigenesis.

Altogether our findings indicate that deletion of MCF2L CRE led to a decrease in *MCF2L* expression, which in turn enhanced the tumorigenic characteristics of SK-N-BE(2) cells. This suggests that *MCF2L* might function as a tumor suppressor gene in this context. Additionally, given the effect of MCF2L CRE SNV1 on MCF2L CRE regulatory activity (Supplementary Figs. 3A and 4A), this variant could contribute to the attenuation of *MCF2L* expression.

### *CTTNBP2* and *MCF2L* are potential tumor suppressors in neuroblastoma

**Fig. 6:**
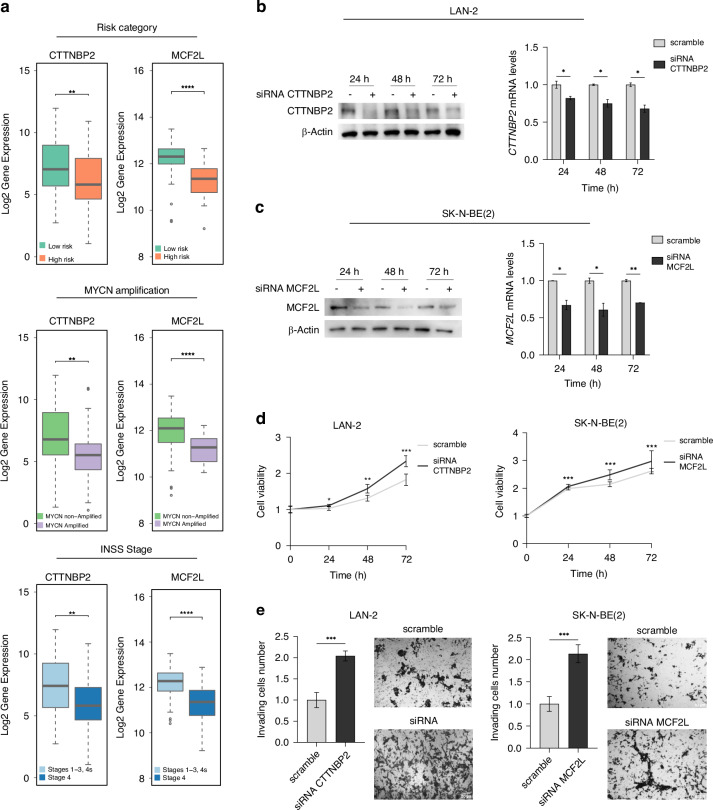
Decreased expression of CTTNBP2 and MCF2L is correlated with more adverse prognostic characteristics and an enhanced tumorigenic phenotype in cells. **a** CTTNBP2 (left) and MCF2L (right) expression by clinical features. From the top, risk group, MYCN amplification and INSS Stage. **b** In LAN-2 cells transfected with three pooled CTTNBP2 siRNA, CTTNBP2 expression relative to siScramble (scramble), as measured by western blot (left) and qPCR (right) 24 h, 48 h and 72 h post CTTNBP2 silencing. **c** SK-N-BE(2) cells transfected with three pooled MCF2L siRNA, MCF2L expression relative to siScramble (scramble), as measured by western blot (left) and qPCR (right) 24 h, 48 h and 72 h post MCF2L silencing. β-Actin protein levels are used as loading control. **d** Cell viability assays in LAN-2 (left) and SK-N-BE(2) (right) cells silenced for CTTNBP2 and MCF2L respectively, are shown as fold change compared to wild-type cells. **e** Invading cells number of LAN-2 (left) and SK-N-BE(2) (right) silenced for CTTNBP2 and MCF2L respectively, are shown as fold change compared to wild-type cells. Representative images (10×) of invasion assays in silenced cells are reported on the right. All data shown are the mean ± standard deviation from three independent qPCR experiments, each done in triplicate. Significant *p*-values obtained by two-tailed T-test are reported by * (* < 0.05; ** < 0.01; *** < 0.001).

In our previous study, we reported an association between low expression levels of *CTTNBP2* and *MCF2L* and unfavorable prognostic indicators in NB [6]. In the current study, we further validate these findings by analyzing an independent dataset comprising mRNA expression data from 105 NB patient samples (GSE73517). Our analysis confirms that lower expression of *CTTNBP2* and *MCF2L* is significantly correlated to worse prognostic features, including the high-risk category (*CTTNBP2*: *p* = 0.009; *MCF2L*: *p* = 2.78 × 10^−10^), presence of *MYCN* amplification (*CTTNBP2*: *p* = 0.003; *MCF2L*: *p* = 1.94 × 10^−6^), and advanced disease stage, specifically stage 4 according to the International Neuroblastoma Staging System (INSS) (*CTTNBP2*: *p* = 0.002; *MCF2L*: *p* = 5.37 × 10^−9^), (Fig. 6a).

These findings support the hypothesis that both *CTTNBP2* and *MCF2L* may function as tumor suppressors in NB. To further confirm this hypothesis, gene silencing experiments were performed in NB cell lines, demonstrating that *CTTNBP2* (Fig. 6b and Supplementary Fig. 9A) or *MCF2L* (Fig. 6c and Supplementary Fig. 9B) silencing led to a significant increase in cell proliferation (Fig. 6d) and invasiveness (Fig. 6f).

Overall, these findings provide strong evidence supporting the role of *CTTNBP2* and *MCF2L* in NB tumorigenesis, suggesting that their reduced expression contributes to the development and progression of more aggressive and high-risk NB phenotypes.

## Discussion

The application of WGS in the exploration of novel somatic drivers of tumorigenesis has unveiled a significant presence of SNVs outside of protein-coding regions in the genome [2]. While traditionally somatic non-coding SNVs were not considered primary contributors to oncogenesis, recent discoveries have highlighted their potential to promote cancer malignant transformation, particularly when they affect functional elements [1–7]. Therefore, the identification of pathogenic drivers within non-coding and regulatory regions is gaining importance, especially for tumors characterized by a low frequency of coding somatic mutations, such as NB [22].

Despite the growing recognition of non-coding regulatory SNVs as potential cancer drivers in computational studies, their biological roles have remained underexplored through in vitro experiments [8]. Consequently, in this study, we conducted a comprehensive investigation of non-coding regulatory SNVs to substantiate their involvement in carcinogenesis and to elucidate the molecular mechanisms through which they may contribute to tumorigenesis.

Our recent findings revealed the presence of putative driver non-coding SNVs within active regulatory elements of NB, which were predicted to impact genes associated with lower survival and worse prognosis in NB patients [6]. Specifically, we identified two SNVs (CTTNBP2 SNV1 and CTTNBP2 SNV2) in a region adjacent to *CTTNBP2*, encoding a neuron-specific cytoskeleton-associated protein involved in neuronal differentiation [35], as well as two SNVs (MCF2L SNV1 and MCF2L SNV2) in an intergenic region predicted to interact with *MCF2L*, codifying a RhoGEF involved in Rho/Rac pathways affecting cytoskeleton dynamics, differentiation, and malignant transformation [36].

In this study, to investigate the potential oncogenic role of these somatic non-coding variants in the initiation and progression of NB, we employed various experimental procedures and functional assays. We observed that CTTNBP2 CRE SNV1 and MCF2L CRE SNV1 reduced the transcriptional activity of their respective regulatory elements. On the other hand, CTTNBP2 CRE SNV2 showed opposite effects in the two investigated NB cell lines. We further investigated the mechanisms by which these SNVs affect gene expression, which plays a pivotal role in tumorigenesis. CTTNBP2 CRE SNV1 and MCF2L CRE SNV1 reduced the binding for the TF STAT3, indicating their role in modulating STAT3-mediated transcriptional regulation. Additionally, CTTNBP2 CRE SNV2 was shown to influence the binding of the TF SIN3A in a cell-specific manner. Together these results underscore the significance of the cellular context and genetic background in determining the impact of non-coding variants. In addition, these findings emphasize that the role of these variants may be dependent upon the expression of TFs and the chromatin changes and accessibility of tissue-specific regulatory elements.

We used CRISPR-Cas9 to generate isogenic cell lines and assess the impact of SNVs on CREs and target genes expression. In this way, we observed that edited clones for both CTTNBP2 and MCF2L CREs led to reduced *CTTNBP2* and *MCF2L* expression levels, respectively, increased cell proliferation and invasiveness, suggesting their role as tumor suppressor. As further evidence of these findings, lower *CTTNBP2* and *MCF2L* expression correlated with poor prognosis markers in NB patients, including high risk*, MYCN* amplification and stage 4.

*CTTNBP2* and *MCF2L* have established roles in neuronal differentiation pathways, and our study sheds light on their potential involvement in NB development. Specifically, CTTNBP2 is known to regulate the subcellular distribution of synaptic proteins, such as cortactin, and play a crucial role in dendritic spine formation and maintenance [35, 37]. Moreover, it has been identified as a significant candidate gene in autism-spectrum disorders, [38–40] with autism-linked mutations in *CTTNBP2* leading to a reduction in dendritic spine density through various mechanisms [41]. Collectively, this evidence underscores the importance of *CTTNBP2* in neuronal differentiation, a critical cellular process often impaired in NB formation [42]. Similarly, *MCF2L* is reported to be associated with autism spectrum disorder, being involved in the control of synapse maturation and, in turn, in neuronal differentiation process [43]. Furthermore, recent research has revealed lower expression levels of *MCF2L* and other genes involved in Rho signal transduction in stage 4 NB patients with unfavorable prognosis compared to those with the same disease stage but a more favorable outcome [44]. Altogether, these findings align with our results, suggesting that *CTTNBP2* and *MCF2L* may contribute to NB development and potentially exhibit tumor-suppressive roles. However, further in vitro and in vivo studies are required to comprehensively validate their biological functions in NB development and progression.

STAT3 and SIN3A also are known to be involved in neuronal functions and development. In detail, *STAT3* has been the subject of extensive research over the past decade to elucidate its significance and role in cancer. While *STAT3* constitutive activation has been linked to the development of several solid cancers, including NB, recent research has also suggested that this TF may function as a tumor suppressor in specific contexts and conditions [45, 46]. Notably, in certain cancer types, STAT3 has shown associations with reduced cancer progression and prolonged survival [47–52]. Consistent with these recent findings, our study highlights a potential role for STAT3 as a tumor suppressor in NB. Additionally, several studies have emphasized the role of *STAT3* in promoting neuronal differentiation [43, 53–56].

The other TF involved in the regulation of *CTTNBP2*, SIN3A, acts as a global transcriptional regulator in different cellular processes, primarily acting as co-repressor [57, 58]. Similarly to STAT3, SIN3A plays a crucial role in neuronal functions and development. It forms a core complex with binding partners involved in neuronal functions, such as MECP2, REST, and coREST [59]. Recent discoveries have linked *SIN3A* mutations to several cases of autism spectrum disorder and mild intellectual disability. *SIN3A* knockdown has also been associated with dysfunctional cortical neuronal development [60].

Considering that NB represent a tumor characterized by impaired neuronal differentiation [42], we can speculate that non-coding variants located in regulatory elements associated with both STAT3 and SIN3A transcriptional regulation could impact neuronal development. Specifically, dysregulation of STAT3 and SIN3A activity during specific developmental stages may inhibit the expression of critical genes involved in differentiation processes, including *CTTNBP2* and *MCF2L*, potentially contributing to NB pathogenesis.

## Conclusions

In summary, our study underscores the importance of employing a functional validation approach to elucidate the biological effects of putative pathogenetic variants in non-coding regulatory elements identified through genome-wide studies. Our approach, which focuses on characterizing both SNVs and their target genes, has proven effective in uncovering novel potential clinically actionable targets that could enhance personalized medicine strategies in the context of NB.

## Supplementary information

Supplementary Information

## Data Availability

RNA-seq data from NB cell lines were obtained from DepMap portal (Expression public 22Q4 release). RNA expression data from NB tumors are available in the Gene Expression Omnibus (GEO) with GSE73517 accession code. No disclosures were reported.
