# Supplementary material for: Regulatory non-coding somatic mutations as drivers of neuroblastoma
Source: Br J Cancer. 2025 Jan 23;132(5):469–80. doi: 10.1038/s41416-025-02939-0 (PMC11876587; doi:10.1038/s41416-025-02939-0)
Supplement: Supplementary file 1 — Supplementary Information [file 41416_2025_2939_MOESM1_ESM.docx]

**Supplementary Information**

**Regulatory non-coding somatic mutations as drivers of neuroblastoma**

Annalaura Montella^1,2*^, Matilde Tirelli^1,2*^, Vito Alessandro Lasorsa^2^, Vincenzo Aievola^2^, Vincenza Cerbone^2^, Rosa Manganiello^2^, Achille Iolascon^1,2^, Mario Capasso^1,2^.

1 University of Naples Federico II, Department of Molecular Medicine and Medical Biotechnology, Naples, Italy

2 CEINGE Biotecnologie Avanzate Franco Salvatore, Naples, Italy

* These authors contributed equally to this work

**Corresponding author:**

Mario Capasso,

University of Naples Federico II

Department of Molecular Medicine and Medical Biotechnology, Naples, Italy

Via Gaetano Salvatore, 486

80145 Napoli Italy

Office: +39 081 37 37 889

Lab: +39 081 37 37 736

Fax +39 081 37 37 804

Supplementary Information contains: the Supplementary Materials section, where the materials and methods of the manuscript are detailed, the Supplementary Figures and the Supplementary Tables section.

**Supplementary Materials**

**Cell culture**

The human LAN-2 (RRID:CVCL_1829) and CHP-212 (RRID:CVCL_1125) were grown in Eagle's Minimum Essential Medium (EMEM)/Nutrient Mixture F-12 (F-12) with non-essential amino acids; SH-SY5Y (RRID:CVCL_0019), SH-EP (RRID: CVCL_0524) and HEK-293 (RRID:CVCL_0045) in Dulbecco’s modified Eagle’s medium (DMEM) and SK-N-BE(2) (RRID:CVCL_0528) in DMEM/ F-12. All growth media were supplemented with 10% heat-inactivated fetal bovine serum (FBS) (Sigma), 1 mM L-glutamine, penicillin (100 U/ml) and streptomycin (100 µg/ml) (Invitrogen). All cells were cultured at 37 °C, under 5% CO2 in a humidified atmosphere. The cell lines were authenticated by STR profiling and tested as mycoplasma-free. Early-passage cells were used for all the experiments.

**Western Blotting**

Antibodies used: anti-CTTNBP2 (17893-1-AP, Proteintech), anti-MCF2L (PA5-22223, Invitrogen), anti-STAT3 (06596, Millipore), anti-SIN3A (ab3479; Abcam) and β-actin (Sigma, A5441). Densitometry analysis was performed quantifying bands intensity by ImageJ software. In brief, the images were converted to 8-bit format and the background was subtracted through the rolling ball radius method. Using rectangular ROI selection and “Gels” function, we selected and circumscribed each band and we quantified peak area of obtained histograms.

**Real-time PCR**

The primer used for Real-time PCR are listed below:

CTTNBP2 For: 5′-GTTTGCCACAAGGGAACAGG-3′;

CTTNBP2 Rev: 5′-GTGACCAGCCTGAGACGTGGC-3′;

MCF2L For: 5′-AAGCCCGGTTATCACCTTCC-3′;

MCF2L Rev: 5′-GGAGGTCCATTTGTCCCGT-3′;

STAT3 For: 5’-CTTTGAGACCGAGGTGTATCACC-3’;

STAT3 Rev: 5’-GGTCAGCATGTTGTACCACAGG-3’;

SIN3A For: 5’-AGCTATCGAGCCTTACCAAAGAGTT-3’;

SIN3A Rev: 5’-GAAGGGAAGGAAACCCAGGTATC-3’;

β-actin For: 5’-CGTGCTGCTGACCGAGG-3’;

β-actin Rev: 5’-GAAGGTCTCAAACATGATCTGGGT-3’;

**Luciferase assays**

To perform luciferase assays. genomic regions corresponding to CREs interacting with CTTNBP2 (CTTNBP2 CRE) and MCF2L (MCF2L CRE) were amplified and directionally cloned upstream of the firefly luciferase gene into the pGL3-Vector and pGL3 -Promoter- Vector plasmid (Promega), respectively. The sequence of each construct was confirmed by direct sequencing. The resulting plasmids containing the CTTNBP2 CRE and MCF2L CRE regions were site-specifically mutated to separately introduce the SNVs (chr7:117513318:G>C or CTTNBP2 CRE SNV1, chr7:117513582:G>T or CTTNBP2 CRE SNV2, chr13:114058179:T>C or MCF2L CRE SNV1 and chr13:114058184:G>A or MCF2L CRE SNV2) using Site-Directed Mutagenesis Kit (Stratagene). Cell lines transfection with luciferase constructs and the analysis on luciferase activity using the Dual-Luciferase Reporter Assay System (Promega) were performed. The same cloning and luciferase assay procedures were performed to obtain shorter constructs (CTTNBP2 CRE ChIP (SNV1) = 229 bp, CTTNBP2 CRE ChIP (SNV2) = 265 bp and MCF2L CRE ChIP which contain both SNV1 and SNV2 of the same regulatory element = 367 bp) which were then used for ChIp-qPCR experiments, as explained subsequently.

Luciferase assays alone and in combination with gene silencing were performed at 24h from plasmid transfection.

PCR primers containing recognition sites for restriction enzymes used to obtain both longer and shorter luciferase assay constructs are:

CTTNBP2 CRE XhoI: 5’-ccgctcgagcggCAAATTCGCCAGGAAACACG-3’;

CTTNBP2 CRE NheI: 5’-aaactagctagctagTGATTCCTCGCGGTGGGATA-3’;

MCF2L CRE NehI: 5’-aaaactagctagctagGAAGGACAAATCGAAGGCCC-3’;

MCF2L CRE XhoI: 5’-ccgctcgagcggGACCCAGATAAAGGCCCAGAAATG-3’;

CTTNBP2 CRE ChIP (SNV1) XhoI: 5’-ccgctcgagcggGTCCCGGGCTTACGGAAC-3’;

CTTNBP2 CRE ChIP (SNV1) NheI: 5’-aaaactagctagctagCCGAGCTCGCATTCGGA-3’;

CTTNBP2 CRE ChIP (SNV2) XhoI: 5’-ccgctcgagcggCTAGCGGATCCGAATGCGAG-3’;

CTTNBP2 CRE ChIP (SNV2) NheI: 5’-aaaactagctagctagATTTCTCGCACCTCCACGAG-3’;

MCF2L CRE ChIP NheI: 5’-aaaactagctagctagGGAGAGGTGGCAATGCTCTT-3’;

MCF2L CRE ChIP XhoI: 5’-ccgctcgagcggCCCACATAGAGGCTCAGGGT-3’;

**ChIP-qPCR experiments**

CTTNBP2 CRE ChIP (SNV1), CTTNBP2 CRE ChIP (SNV2) and MCF2L CRE ChIP plasmids containing the wild-type and the mutant sequence were transfected into NB cells using X-tremeGENE HP DNA Transfection Reagent (Roche) per manufacturer’s instruction. Next day, each STAT3 and SIN3A antibodies used for western blotting were conjugated with 10 µL of each Dynabeads A and G (ThermoFisher Scientific) for each ChIP for 6h with rotation at 4 °C, at concentration of 5 µg. After 24h from transfection, cells were fixed by resuspending with 300 µL of 1% formaldehyde in PBS for 10 min at room temperature and a final concentration of Glycine was added to quench excess formaldehyde. Cells were then lysed using the Modified RIPA buffer (10 mM TrisHCl, pH 8.0; 1 mM EDTA; 140 mM NaCl; 1% Triton X-100; 0.1% SDS; 0.1% sodium deoxycholate) supplemented with protease inhibitor. The lysates were subject to 30 cycles of sonication (30 s ON 30 s OFF, high frequency) using Diagenode Bioruptor Plus (Diagenode). After collecting 15 µL of sonicated lysate as input, the samples were incubated with antibody beads conjugates overnight incubation at 4 °C with rotation. Next day, the beads were washed and decrosslinking was performed for 6h at 65 °C. Samples were then purified and eluted. ChIP and input DNA were then used for allele-specific ChIP-qPCR using primers designed and listed below. Fold-change significance was calculated using Student’s t test and all analyses were done using hg19 reference genome coordinates.

Primer list:

MAMA Primer CTTNBP2 CRE ChIP (SNV1) wt seq: 5’-CCGCCGCGGCCCCAGGGTCG-3’

MAMA Primer CTTNBP2 CRE ChIP (SNV1) mut seq: 5’-CCGCCGCGGCCCCAGGGTGC-3’

MAMA Primer CTTNBP2 CRE ChIP (SNV2) wt seq: 5’-CTCACGGCGCCGAGGGCCGAGGG-3’

MAMA Primer CTTNBP2 CRE ChIP (SNV2) mut seq: 5’-CTCACGGCGCCGAGGGCCGAGCT-3’

MAMA Primer MCF2L CRE ChIP wt seq: 5’-CCATCCCACTTGTCAAGGCCTGT-3’

MAMA Primer CTTNBP2 CRE ChIP mut seq: 5’-CCATCCCACTTGTCAAGGCCTCC-3’

GLprimer2_R: 5’-CTTTATGTTTTTGGCGTCTTCCA-3’

PGL3-Promoter-Vector_CostantPrimer: 5’-GGGGCGGGACTATGGTTGCT-3’

**CRISPR-Cas9 experiments**

To obtain INDEL mimicking CTTNBP2 CRE SNV1, two guide RNAs (gRNA1 CTTNBP2: 5’-GGTGCGAAGTCGGGTCCGGC-3’ and gRNA2 CTTNBP2: 5’-GTGCGAAGTCGGGTCCGGCT-3’) were designed with CRISPOR Tool (http://crispor.tefor.net/), cloned into pSpCas9(BB)-2A-GFP (PX458) vector expressing Cas9 (Addgene) and used for this editing experiments. After 48h from transfection, GFP-positive cells were sorted at single cell levels into a 96 well. Genome editing was validated by PCR followed by Sanger sequencing. In particular, PCR was performed by using KAPA HiFi HotStart PCR Kit (Roche) with the following primer pairs: Primer Forward: 5’- tgtaaaacgacggccagtCGGAAGGGACAGGAGGGAT -3’ and Primer Reverse: 5’- caggaaacagctatgaccCCGCTAGAGCAGGAAGATGG -3’ (where M13 sequence is highlighted in lower case). An analysis on electropherograms using the web tool synthego (ice.synthego.com) revealed the successful editing.

To delete the region containing both MCF2L CRE SNV1 and MCF2L CRE SNV2, two gRNAs upstream the region (gRNA1 MCF2L: 5’-GACAAGTGGGATGGCGCCTC3- and gRNA2 MCF2L: 5’-GACAAGTGGGATGGCGCCTC-3’) each one combined with a gRNA downstream (gRNA3 MCF2L: 5’-TCGGGAGCAGACCCACATAG -3’) were used. gRNAs were cloning in PX458 vector as described above and resulted constructs were cotransfected into SK-N-BE(2) cells. To identify and distinguish both mono-allelic and bi-allelic deletions, a PCR using two primer pairs flanking the sgRNA cleavage sites (Primer-OUT-Forward: 5′- CAATGGGCGGATGTCACAGT-3′; Primer-OUT-Reverse: 5′-GGAACGGGTTCAGGGTCA-3′) was performed with KAPA HiFi HotStart PCR Kit (Roche), following manufacturer’s instructions.

**Statistical analysis**

All experiments were repeated on at least three occasions, in triplicate. The means and standard deviations were calculated from three independent experiments and the differences among groups were analyzed using unpaired student’s t-test. p-value < 0.05 were considered statistically significant. *p-value ≤ 0.05, **p-value ≤ 0.01, ***p-value ≤ 0.001.

**Supplementary Figures**


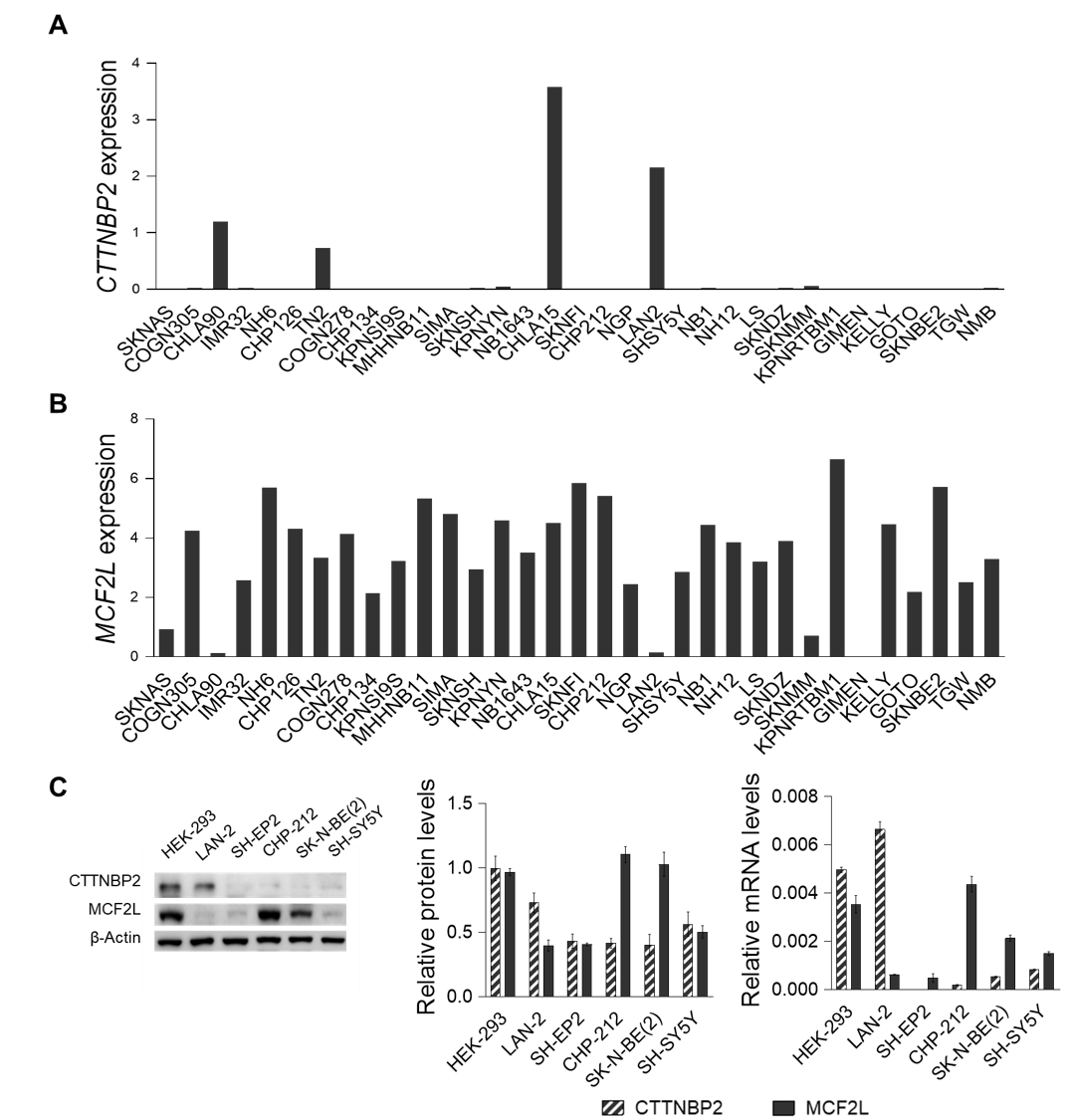


**Supplementary Figure 1: CTTNBP2 and MCF2L expression levels across NB cell lines. (A)** CTTNBP2 and **(B)** MCF2L mRNA expression levels across 33 NB cell lines are obtained from the DepMap portal (https://depmap.org/portal/) (Expression public 20Q2) and expressed as Log2 (TPM+1). **(C)** CTTNBP2 and MCF2L protein levels are evaluated by western blot and densitometry (left), while mRNAs levels are assessed by qPCR (right) in available NB cell lines. β-Actin protein levels are used as loading control. Data shown are the mean ± standard deviation from three independent qPCR experiments, each done in triplicate.

**
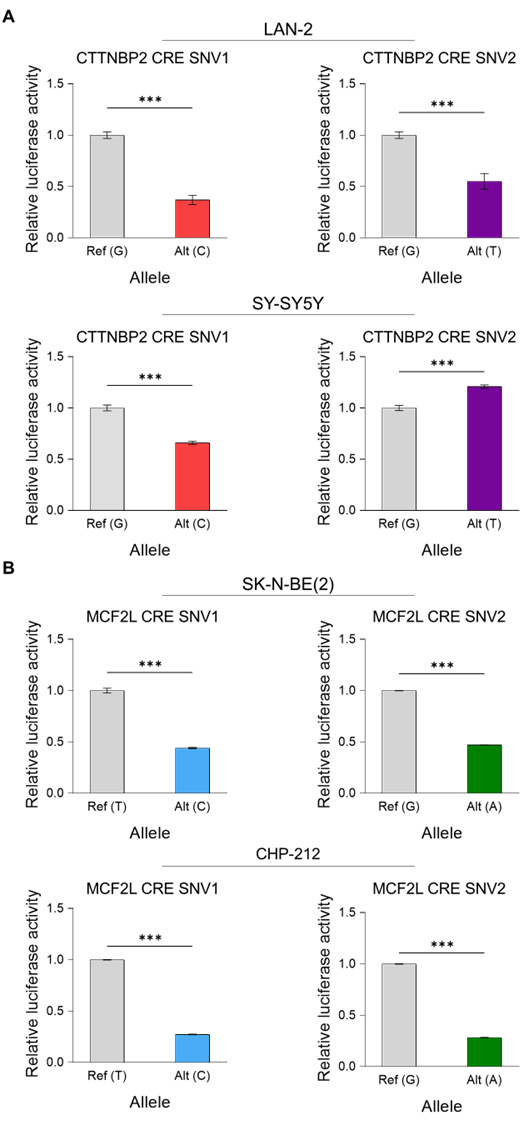
**

**Supplementary Figure 2: Somatic non-coding SNVs affect the transcriptional activity of CTTNBP2 and MCF2L CREs.** Luciferase reporter gene assays carried out in **(A)** LAN-2 and SH-SY5Y for CTTNBP2 CRE SNV1 (in red) and CTTNBP2 CRE SNV2 (in purple) and in **(B)** SK-N-BE(2) and CHP-212 for MCF2L CRE SNV1 (in light blue) and MCF2L CRE SNV2 (in green). Luciferase activity of CTTNBP2 CRE (944 bp) and MCF2L CRE (1000 bp) is normalized to the internal Renilla control, after subtraction of pGl3 Basic Vector and pGl3 Promoter Vector activity, respectively. The datapoints represent the mean of technical triplicates of each of the three independent experiments. Significant p-values obtained by two-tailed T-test are reported by *(* <0.05; ** <0.01; *** <0.001). Ref: Reference; Alt: Altered.

**
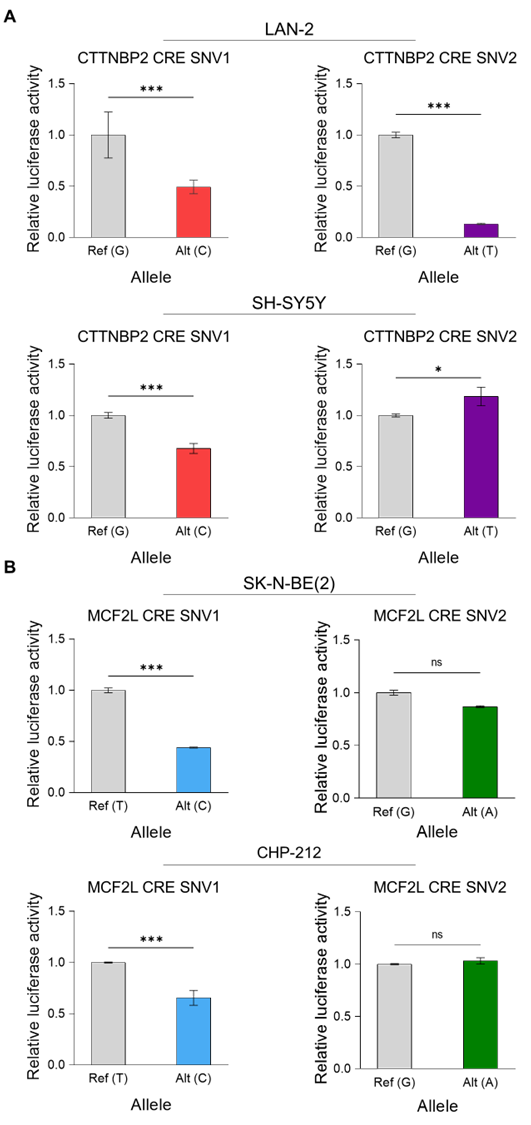
**

**Supplementary Figure 3: Luciferase assays conducted on shorter constructs confirmed the alteration of** **transcriptional activity of CREs due to the presence of CTTNBP2 CRE SNV1, SNV2, and MCF2L CRE SNV1.** Luciferase reporter gene assays carried out (**A**) in LAN-2 and SH-SY5Y for CTTNBP2 CRE SNV1 (in red) and CTTNBP2 CRE SNV2 (in purple) and (**B**) in SK-N-BE(2) and CHP-212 for MCF2L CRE SNV1 (in light blue) and MCF2L CRE SNV2 (in green). Luciferase activity of CTTNBP2 CRE SNV1 (229 bp) and CTTNBP2 CRE SNV2 (265 bp) constructs is relative to PGL3 empty vector, while that of MCF2L CRE SNV1 and SNV2 (367 bp) construct is related to PGL3 promoter vector. The datapoints represent the mean of technical triplicates of each of the three independent experiments. Significant p-values obtained by two-tailed T-test are reported by * (* <0.05; ** <0.01; *** <0.001). Ns: non-significant p-value; Ref: Reference; Alt: Altered.

**
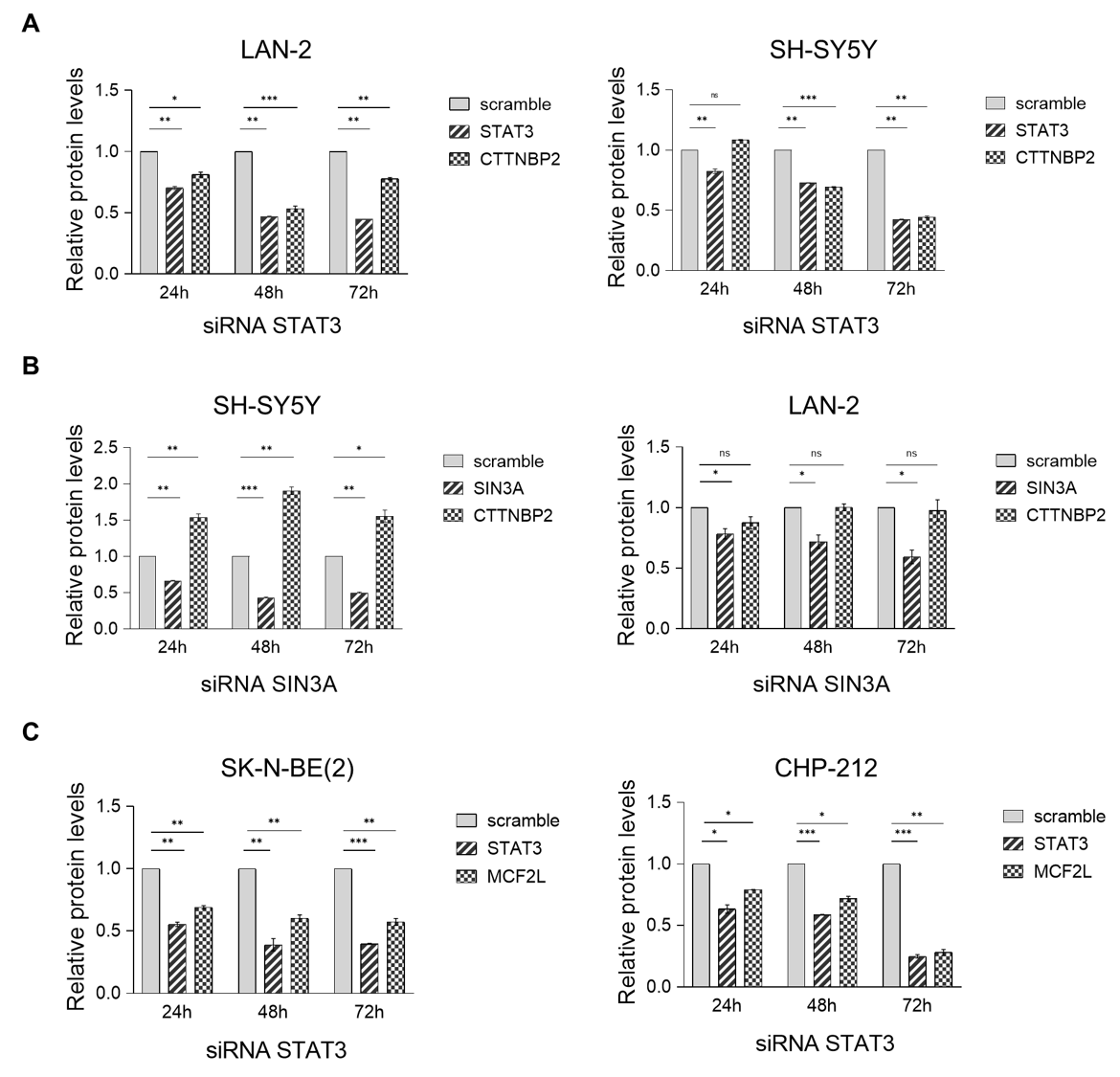
**

**Supplementary Figure 4: Densitometry analyses showed a correlation between protein levels of a TF and its target gene.** Correlations between STAT3 and CTTNBP2 (**A**), SIN3A and CTTNBP2 (**B**), STAT3 and MCF2L (**C**) were evaluated by measuring protein levels from their respective Western Blotting. The datapoints represent the mean of technical triplicates of each of the three independent experiments and they are presented as fold-change on siScramble (scramble). Significant p-values obtained by two-tailed T-test are reported by * (* <0.05; ** <0.01; *** <0.001). Ns: non-significant p-value; Ref: Reference; Alt: Altered.


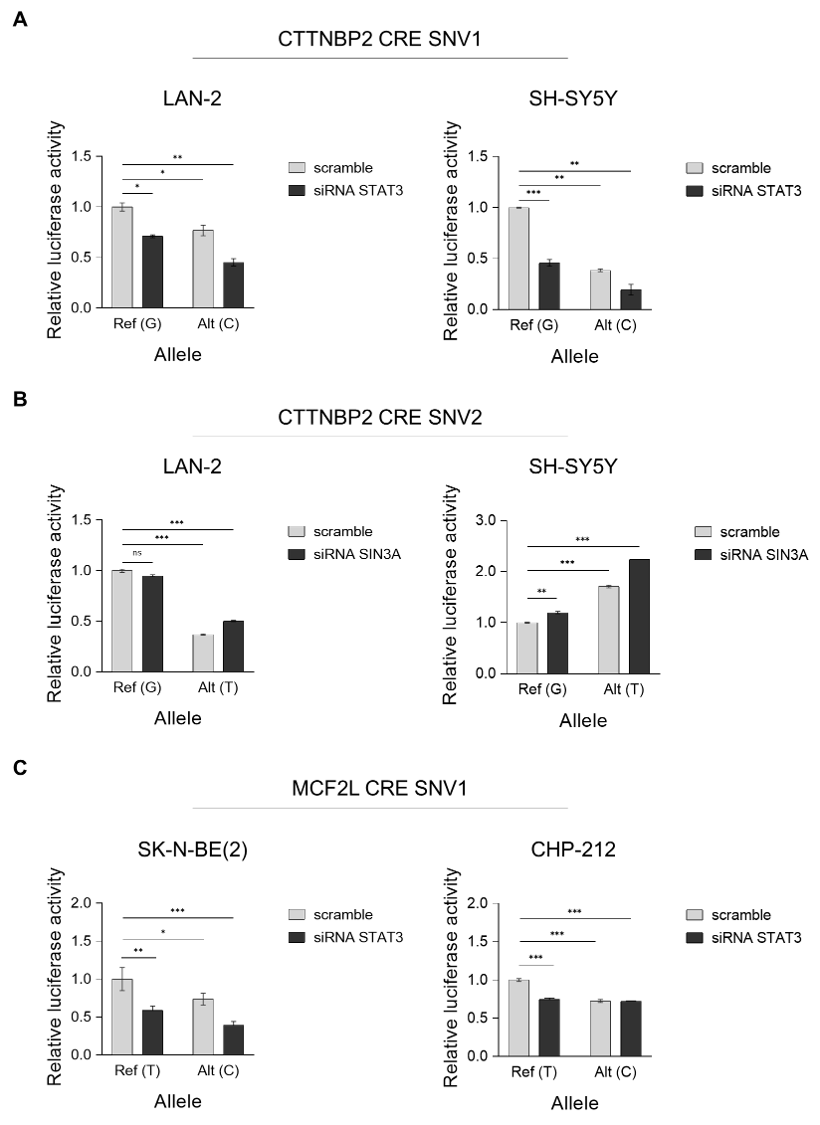


**Supplementary Figure 5: Luciferase assays on shorter constructs in combination with TFs silencing confirm the binding alteration caused by the presence of SNVs.** Luciferase reporter gene assays for (**A**) CTTNBP2 CRE SNV1 and (**B**) CTTNBP2 CRE SNV2 are performed in both LAN-2 (left) and SH-SY5Y (right) silenced by STAT3 and SIN3A, respectively. (**C**) The same experiment is performed in SK-N-BE(2) and CHP-212 72h post STAT3 silencing to evaluate regulatory activity of MCF2L CRE SNV1. Luciferase activity of non-coding variants is normalized to that from cells transfected with wild type construct and siScramble (scramble). The datapoints represent the mean of technical triplicates of each of the three independent experiments. Significant p-values obtained by two-tailed T-test are reported by * (* <0.05; ** <0.01; *** <0.001). Ns: non-significant p-value; Ref: Reference; Alt: Altered.

**
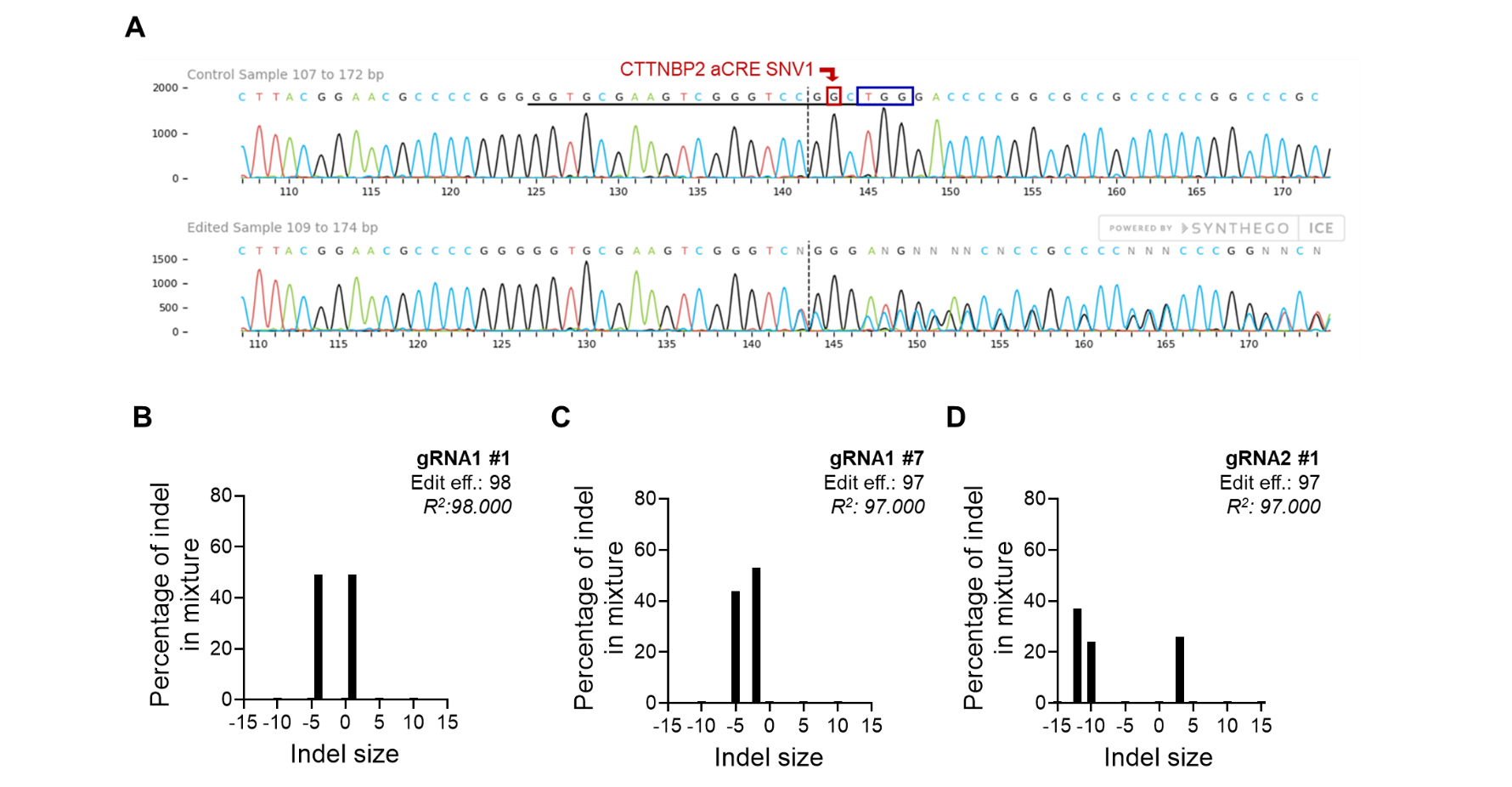
**

**Supplementary Figure 6. Genome editing results of CTTNBP2 CRE SNV1** (**A**) Representative electropherogram of edited clones. Sanger sequencing traces showing the Cas9 cutting site in proximity of CTTNBP2 CRE SNV1 (top line) and subsequent waveform decomposition in edited cells. Black line: gRNA sequence; blue box: PAM sequence. (**B-D**) Bar plots representing the INDELs distribution in HEK-293 gRNA1 #1, gRNA1 #7 and gRNA2 #1 edited clones. (**B**) The gRNA1#1 clone exhibits a 4 bp deletion (chr7:117513316-117513319:CGGC:-) with an indel frequency of 49%, and a concurrent 1 bp insertion (chr7:117513316-117513317:CG:CNG) at the same frequency, cumulatively achieving a 98% editing efficiency. (**C**) The second clone, gRNA1#7, had a 2 bp deletion (chr7:117513317-117513318:GG:-) with 53% indel frequency, coexisting in heterozygosity with a 5 bp deletion (chr7:117513317-117513321:GGCTG:-) at a 44% indel frequency, totalling a 97% editing efficiency. (**D**) The third clone, gRNA2#1, featured different INDELs including the deletion of the site encompassing CTTNBP2 CRE SNV1 in 68% of the cell population, resulting in a total editing efficiency of 97%.

**
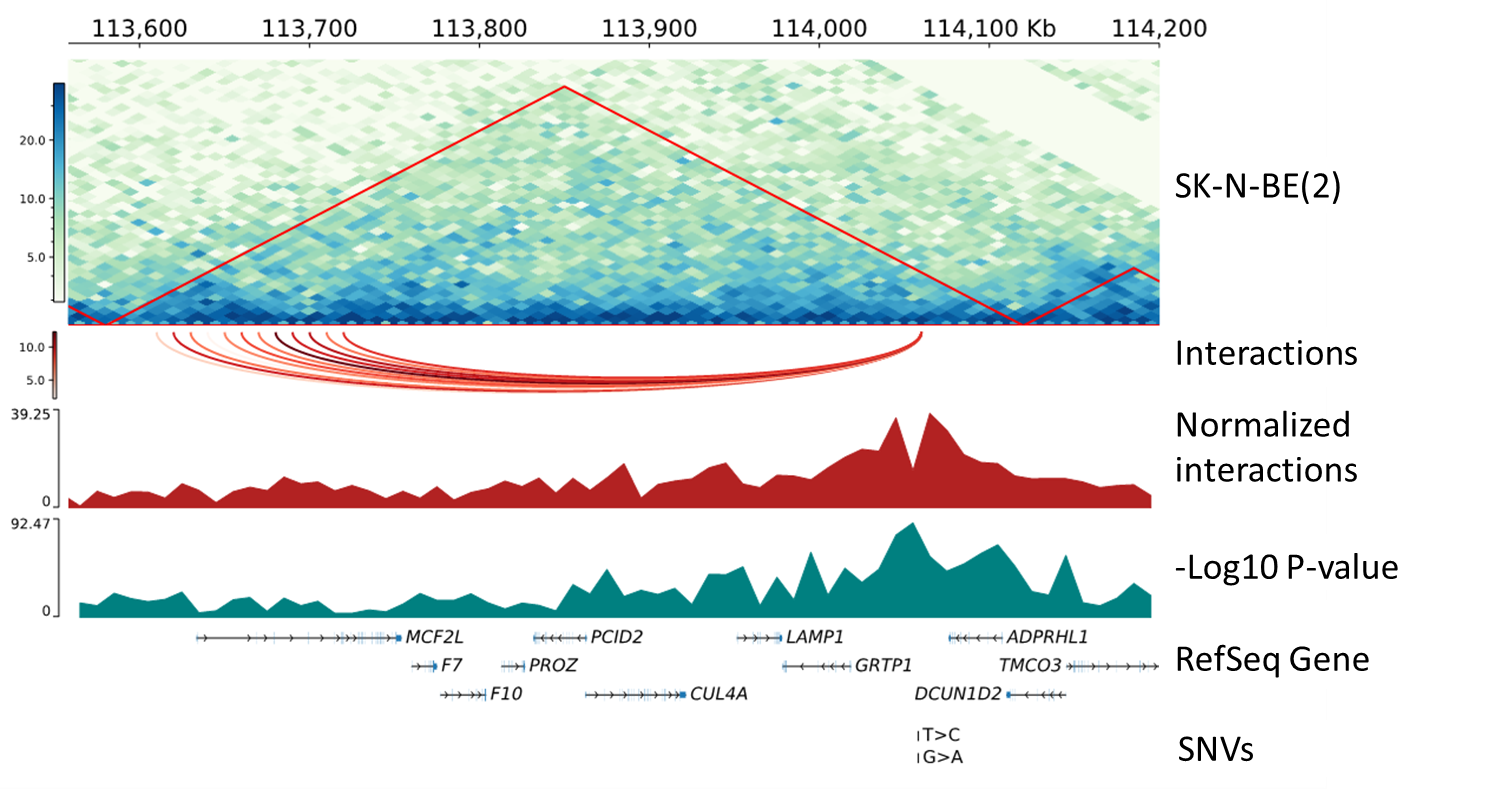
Supplementary Figure 7. Interaction between *MCF2L* and MCF2L CRE.** The figure reports the genomic interactions of MCF2L CRE. The genomic tracks are named from top to bottom and described below. The genomic coordinates are from human genome hg19. The interaction matrix is centred on cluster variants’ genomic coordinates (chr13:114058178-114058287) and extended of 0.5 Mb up- and downstream. Genomic coverage is of 600 Kb and the matrix resolution is of 10 Kb (the interactions are calculated between bins of 10 Kb). Red-bordered triangles represent the Topologically Associated Domains (TADs). The arcs track shows the interactions between MCF2L CRE and *MCF2L* annotated bins. The normalized number of interactions (a measure of the strength of interactions). The minus Log10 of the FDR adjusted p value. The NCBI RefSeq genes are reported.

**
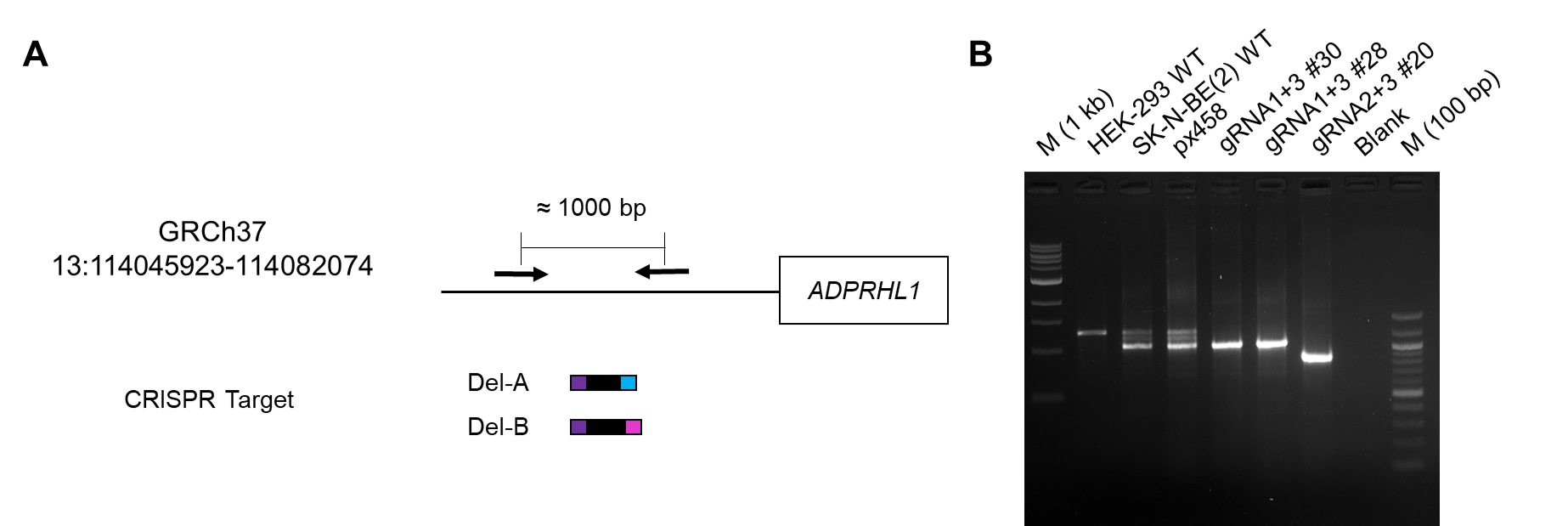
**

**Supplementary Figure 8. Genome editing results about MCF2L CRE.** (**A**) Schematic representation of CRISPR-Cas9 strategy in SK-N-BE(2) targeting MCF2L CRE. In the top the genomic region containing the regulatory element and PCR primers positions (black arrows) are reported. At the bottom, regions targeted for deletion are represented by the black bars flanked by the pairs of single guide RNAs (purple, light blue and pink). (**B**) Visualization of PCR products on agarose gel to screen edited clones. HEK-293 (lane 2), SK-N-BE(2) wild-type (lane 3) and SK-N-BE(2) transfected with pX458 vector (lane 4) cells are used as wild-type controls. The three bands exhibited by both SK-N-BE(2) controls correspond to our target region. In lanes 5 and 6, RNA 1+3#30 and gRNA 1+3#38 show a single amplicon in contrast to control SK-N-BE(2) cells. Inferring that our target region is effectively removed in at least a single allele, RNA 1+3#30 and gRNA 1+3#38 constitute heterozygous edited clones. The third edited clone gRNA 2+3#20 (lane 7) lacks any of the three PCR products present in control SK-N-BE(2) cells and exhibit a single, shorter amplicon, indicative of the target region deletion. For this reason, it is considered as homozygous clone. 1 kb (lane 1) and 100 bp (lane 9) markers (M) are used for PCR amplicons length controls.


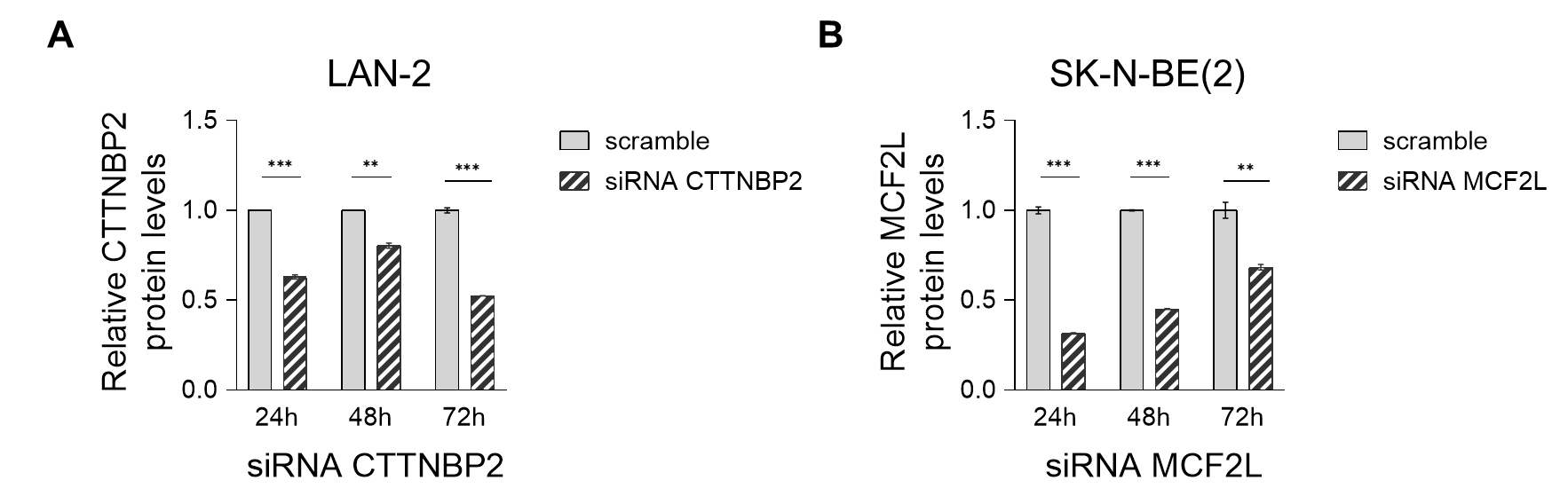


**Supplementary Figure 9: Densitometry analyses showed the effects of CTTNBP2 and MCF2L silencing on their protein levels.** Protein levels of CTTNBP2 (**A**) and MCF2L (**B**) were evaluated by measuring protein levels from their respective Western Blotting. The datapoints represent the mean of technical triplicates of each of the three independent experiments and they are presented as fold-change on siScramble (scramble). Significant p-values obtained by two-tailed T-test are reported by * (* <0.05; ** <0.01; *** <0.001). Ns: non-significant p-value; Ref: Reference; Alt: Altered.

**Supplementary Tables**

**Supplementary Table 1.** TFs motif binding predicted to be altered by chr7:117513318:G>C

| **Variant ID** | **Reference Allele** | **Altered Allele** | **Motif Position** | **Gene Symbol** | **Sequence Match** | **pctRef** | **pctAlt** | **allele Ref** | **alleleAlt** | **effect** | **type** |
| --- | --- | --- | --- | --- | --- | --- | --- | --- | --- | --- | --- |
| chr7:117513318:G>C | G | C | 8 | MBD2 | gggtccgGctg | 0.95 | 0.78 | 1.00 | 0.00 | strong | break |
|  |  |  | 2 | MECP2 | gtccgGc | 0.91 | 0.71 | 1.00 | 0.00 | strong | break |
|  |  |  | 8 | STAT3 | gggtccgGctggg | 0.95 | 0.84 | 0.82 | 0.00 | strong | break |
|  |  |  | 3 | TFCP2 | cgGctgg | 0.93 | 0.75 | 1.00 | 0.00 | strong | break |
|  |  |  | 4 | USF2 | cgGctg | 0.74 | 0.96 | 0.00 | 1.00 | strong | gain |
|  |  |  |  | | | | | | | | |
|  |  |  | 7 | NANOG | ccgGctggga | 0.93 | 0.86 | 0.68 | 0.16 | weak | break |
|  |  |  | 4 | SIN3A | ccgGctgggacc | 0.91 | 0.80 | 0.75 | 0.25 | weak | break |
|  |  |  | 8 | TAF1 | cgGctgggac | 0.91 | 0.81 | 0.72 | 0.20 | weak | break |

**pctRef:** the score as determined by the scoring method, when the sequence contains the reference variant allele, normalized to a scale from 0 - 1.

**pctAlt:** the score as determined by the scoring method, when the sequence contains the alternate variant allele, normalized to a scale from 0 - 1.

**alleleRef:** the proportional frequency of the reference allele at position motifPos in the motif

**alleleAlt:** the proportional frequency of the alternate allele at position motifPos in the motif

**Supplementary Table 2.** TFs motif binding predicted to be altered by chr7:117513582:G>T

| **Variant ID** | **Reference Allele** | **Altered Allele** | **Motif Position** | **Gene Symbol** | **Sequence Match** | **pctRef** | **pctAlt** | **alleleRef** | **alleleAlt** | **effect** | **type** |
| --- | --- | --- | --- | --- | --- | --- | --- | --- | --- | --- | --- |
| chr7:117513582:G>T | G | T | 4 | CHD2 | gccGggagccg | 0.95 | 0.81 | 0.72 | 0.00 | strong | break |
|  |  |  | 4 | ELF1 | gccGggagcc | 0.92 | 0.76 | 0.79 | 0.00 | strong | break |
|  |  |  | 4 | MECP2 | gccGgga | 0.94 | 0.74 | 1.00 | 0.00 | strong | break |
|  |  |  | 7 | POU2F2 | ggagccGggagcc | 0.91 | 0.77 | 0.74 | 0.00 | strong | break |
|  |  |  | 7 | SIN3A | ggagccGggagc | 0.91 | 0.74 | 0.72 | 0.00 | strong | break |
|  |  |  |  | | | | | | | | |
|  |  |  | 3 | NR3C1 | ccGggagccgggag | 0.92 | 0.87 | 0.45 | 0.00 | weak | break |

**pctRef:** the score as determined by the scoring method, when the sequence contains the reference variant allele, normalized to a scale from 0 - 1.

**pctAlt:** the score as determined by the scoring method, when the sequence contains the alternate variant allele, normalized to a scale from 0 - 1.

**alleleRef:** the proportional frequency of the reference allele at position motifPos in the motif

**alleleAlt:** the proportional frequency of the alternate allele at position motifPos in the motif

**Supplementary Table 3.** TFs motif binding predicted to be altered by chr13:114058179:T>C

| **Variant ID** | **Reference Allele** | **Altered Allele** | **Motif Position** | **Gene Symbol** | **Sequence Match** | **pct Ref** | **pctAlt** | **alleleRef** | **alleleAlt** | **effect** | **type** |
| --- | --- | --- | --- | --- | --- | --- | --- | --- | --- | --- | --- |
| chr13:114058178:T>C | T | C | 8 | CHD2 | ctgTtcccgac | 0.66 | 0.94 | 0.00 | 1.00 | strong | gain |
|  |  |  | 5 | MYB | gcctgTtccc | 0.91 | 0.65 | 1.00 | 0.00 | strong | break |
|  |  |  | 3 | MYB | ggcctgTtc | 0.94 | 0.67 | 1.00 | 0.00 | strong | break |
|  |  |  | 9 | MYB | aaggcctgTtc | 0.91 | 0.67 | 1.00 | 0.00 | strong | break |
|  |  |  | 4 | MYBL2 | ggcctgTtcc | 0.93 | 0.71 | 1.00 | 0.00 | strong | break |
|  |  |  | 11 | NFE2 | Ttcccgaccct | 0.79 | 0.91 | 0.00 | 0.71 | strong | gain |
|  |  |  | 4 | NR3C1 | ctgTtcc | 0.94 | 0.75 | 0.93 | 0.04 | strong | break |
|  |  |  | 5 | NR3C1 | cctgTtcc | 0.92 | 0.72 | 0.95 | 0.05 | strong | break |
|  |  |  | 4 | PGR | ctgTtcc | 0.93 | 0.71 | 1.00 | 0.00 | strong | break |
|  |  |  | 4 | STAT1 | ctgTtccc | 0.91 | 0.59 | 0.96 | 0.00 | strong | break |
|  |  |  | 4 | STAT1 | ctgTtccc | 0.92 | 0.71 | 0.94 | 0.02 | strong | break |
|  |  |  | 4 | STAT3 | ctgTtccc | 0.99 | 0.73 | 0.88 | 0.04 | strong | break |
|  |  |  | 4 | STAT5A | ctgTtccc | 0.95 | 0.65 | 1.00 | 0.00 | strong | break |
|  |  |  | 4 | STAT6 | ctgTtccc | 0.92 | 0.65 | 1.00 | 0.00 | strong | break |
|  |  |  | 6 | ZNF263 | gcctgTtccc | 0.79 | 0.95 | 0.00 | 1.00 | strong | gain |
|  |  |  |  |  |  |  |  |  |  |  |  |
|  |  |  | 4 | AR | gcctgTtcc | 0.88 | 0.95 | 0.27 | 0.67 | weak | gain |
|  |  |  | 2 | ETS1 | gTtccc | 0.94 | 0.84 | 0.58 | 0.00 | weak | break |
|  |  |  | 9 | IKZF2 | ctgTtcccgacc | 0.92 | 0.87 | 0.50 | 0.03 | weak | break |
|  |  |  | 7 | ZNF143 | cctgTtcccga | 0.78 | 0.90 | 0.00 | 0.65 | weak | gain |

**pctRef:** the score as determined by the scoring method, when the sequence contains the reference variant allele, normalized to a scale from 0 - 1.

**pctAlt:** the score as determined by the scoring method, when the sequence contains the alternate variant allele, normalized to a scale from 0 - 1.

**alleleRef:** the proportional frequency of the reference allele at position motifPos in the motif

**alleleAlt:** the proportional frequency of the alternate allele at position motifPos in the motif
